# Normal values of wall shear stress in the pulmonary artery from 4D flow data

**DOI:** 10.1186/1532-429X-14-S1-W66

**Published:** 2012-02-01

**Authors:** Julio A Sotelo, Pablo Bächler, Steren Chabert, Daniel Hurtado, Pablo Irarrazaval, Cristian Tejos, Sergio Uribe

**Affiliations:** 1 Biomedical Engineering, Universidad de Valparaíso, Valparaíso, Chile; 2 Biomedical Imaging Center, Pontificia Universidad Católica de Chile, Santiago, Chile; 3 School of Medicine, Pontificia Universidad Católica de Chile, Santiago, Chile; 4 Structural Engineering, Pontificia Universidad Católica de Chile, Santiago, Chile; 5 Electrical Engineering, Pontificia Universidad Católica de Chile, Santiago, Chile; 6 Radiology, Pontificia Universidad Católica de Chile, Santiago, Chile

## Background

Quantification of Wall Shear Stress (WSS) from 4D flow data in the aorta has been recently reported (1). However, this phenomenon has not been extensively studied in the pulmonary artery. The objective of this work was to develop a novel method to calculate WSS using 4D flow data. The method was applied to calculate WSS values in the main, right and left Pulmonary Artery (PA, RPA and LPA).

## Methods

4D flow data of the whole heart (spatial resolution of 2.5m^3^, temporal resolution of 38ms) was acquired on 17 volunteers and 5 patients (1 repaired transposition of the great arteries, 2 after Glenn procedure and 2 with partial anomalous pulmonary vein return (1 patient with Atrial Septal Defect)).

Using a in-house code, three slices were reformatted perpendicular to the main PA, right pulmonary artery (RPA) and left pulmonary artery (LPA). Subsequently, we segmented the blood pool, and calculated Magnitude (WSS-M), Axial (WSS-A), and Circumferential (WSS-C) WSS using a Strain Rate Tensor based on cylindrical coordinates. For each slice, we generated three contiguous slices to include variation of the velocity along the direction of the vessel.

To evaluate the reproducibility of the proposed method, two independent observers analyzed the data.

## Results

Average WSS-M in volunteers was: MPA = 0.17±0.02N/m^2^; RPA = 0.22±0.05N/m^2^; LPA = 0.11±0.01N/m^2^. For patients, the average WSS-M was: MPA = 0.34±0.18N/m^2^; RPA = 0.36±0.1N/m^2^; LPA = 0.28±0.14N/m^2^. Figure 1 shows the average WSS-M calculated along the cardiac cycle for each segment. Figure 2 depicts Bland Altman plots of the mean WSS measured by the two observers, showing a small bias and standard deviations (mean difference of 0.016N/m^2^, 0.008N/m^2^ and 0.005N/m^2^ for the WSS-M in the PA, RPA and LPA respectively).

## Conclusions

In this work we proposed a novel and reproducible method to calculate WSS derived from 4D flow data in the main PA, RPA and LPA. In volunteers, we found a greater WSS in the RPA compared with the LPA, which is probably associated with more complex flow patterns (helices) in the RPA (2). Values of WSS obtained in patients showed increasing values of WSS, probably owe to complex and retrograde flow patterns in the pulmonary circulation.

## Funding

Fondecyt-11100427, Anillo ACT-079.
